# Influence of Alternative Lifestyles on Antibiotic Use during Pregnancy, Lactation and in Children

**DOI:** 10.3390/antibiotics10070837

**Published:** 2021-07-09

**Authors:** Pien Eras, Ana Paula Simões-Wüst, Carel Thijs

**Affiliations:** 1 Department of Epidemiology, CAPHRI Care and Public Health Research Institute, Maastricht University, 6200 Maastricht, The Netherlands; pieneras24@gmail.com (P.E.); c.thijs@maastrichtuniversity.nl (C.T.); 2 Clinic Arlesheim, Research Department, 4144 Arlesheim, Switzerland; 3 University Hospital Zurich, Department of Obstetrics, University of Zurich, 8091 Zurich, Switzerland

**Keywords:** restricted antibiotics use, pregnancy, lactation, children, lifestyle, alternative, anthroposophic medicine

## Abstract

Alternative lifestyles are likely to be associated with distinct usage of specific medicinal products. Our goal was to find out whether the intake of antibiotics during pregnancy and by children differs according to whether the mothers have alternative or conventional lifestyles. Therefore, we investigated the use of antibiotics by pregnant women and by children up to 11 years of age participating in the KOALA Birth Cohort Study. This cohort comprises two recruitment groups of mother–infant pairs, one with alternative lifestyles (selected via organic food shops, anthroposophic clinicians and midwives, anthroposophic under-five clinics, Rudolf Steiner schools and relevant magazines, *n* = 491) the other with conventional lifestyles (no selection based on lifestyle, *n* = 2343). Mothers in the alternative lifestyle group more frequently adhered to specific living rules and identified themselves with anthroposophy more than mothers in the conventional lifestyle group. The results revealed significant differences in antibiotic use during pregnancy and in children from 3 months to 10 years of age between the two groups. The rate of antibiotic use in children was consistently lower in the alternative lifestyle group than in the conventional lifestyle group. Antibiotic use in pregnancy was higher in low educated women, and maternal antibiotic use during lactation was higher after an instrumented delivery in hospital. Antibiotic use in the infant was higher when they had older sibs or were born in hospital, and lower in those who had been longer breastfed. After adjustment for these factors, the differences in antibiotic use between the alternative and conventional groups remained. The results suggest that an alternative lifestyle is associated with cautious antibiotic use during pregnancy, lactation and in children.

## 1. Introduction

Lifestyles perceived to be distinct from those of the majority of people are often called “alternative lifestyles”. Such lifestyles are frequently supported by underlying ideologies and may influence daily practical decisions, such as those concerning school choice for children, diet composition and use of conventional or complementary medicine therapies and medications. Whereas medications from complementary medicine are likely to be frequently used, conventional medications, especially if perceived as problematic, are expected to be avoided.

Antibiotics are among the most useful but also most problematic medications. Whereas their use in the treatment of bacterial infections has had a markedly favorable impact on public health, this success is clouded by the worryingly increasing spread of antibiotic resistance. According to the WHO (see, e.g., [1]), antibiotic resistance is a major public health problem worldwide, to a large extent caused by the inappropriate and excessive use of antibiotics. Several studies show that despite recommendations for rational antibiotic use, their consumption is still higher than ideal (see [2] for an example). Therefore, measures to reduce unnecessary use of antibiotics are urgently needed.

Antibiotics are the most frequently used medication among children worldwide [3,4,5,6,7]. Antibiotic use in children may not only contribute to spreading antibiotic resistance but also affect children’s health. Previous studies have shown that antibiotic exposure early in life can affect weight development in children [8,9,10]. Repeated use of antibiotics, particularly broad-spectrum antibiotics, in children under 24 months of age also seems to be a risk factor for developing childhood obesity [8]. Moreover, exposure to antibiotics before the age of 6 months, or repeatedly during childhood, has been associated with increased body weight in healthy children [9].

The KOALA Birth Cohort Study is a prospective cohort study of mother–infant pairs in the Netherlands with two distinct recruitment groups: one with conventional lifestyles and the other with alternative lifestyles [11]. The aim of the present analysis of KOALA data was to find out whether the intake of antibiotics by children differs according to whether the mothers have alternative or conventional lifestyles. The results might not only motivate pediatricians to better consider antibiotic prescriptions for children and how to deal with parents’ expectations but also alert them to the need for lifestyle-tailored use recommendations.

## 2. Results

### 2.1. Demographic, Lifestyle and Health-Related Characteristics

As depicted in Table 1, mothers of the alternative lifestyle group were on average 2 years older at delivery (33.7 vs. 31.7 years) and more often had a higher education (72.7% vs. 42.5%).

There were several indications that women in the alternative lifestyle group had lifestyles that differed from the conventional one. First, they more often adhered to certain living rules (e.g., those concerning vegetarianism (16.9% vs. 1.5%) and anthroposophy (14.3% vs. 0.1%)) than the women in the conventional lifestyle group. Secondly, they more often actively chose to consume organic food (87.3% vs. 20.8%) and biodynamic products (73.2% vs. 7.2%).

From the beginning of the pregnancy until week 34, a higher percentage of women in the alternative group reported genital Candida infections (22.7% vs. 14.1%) and oral aphthae (12.9% vs. 8.0%) than the conventional group. After the third month of pregnancy, a higher percentage of women in the conventional lifestyle group reported diarrhea and urinary infections (see Table 2). Similar frequencies between the two recruitment groups were found concerning the frequency of Candida infections in the mouth (during the pregnancy), as well as fever and flu (after the third month of pregnancy).

Pregnancy duration was similar in the two groups (39.6 vs. 39.5 weeks), whereas neonates in the alternative group were on average slightly though statistically significantly heavier at birth than those of the conventional group (3582 vs. 3487 g).

### 2.2. Antibiotic Use

**Figure 1:**
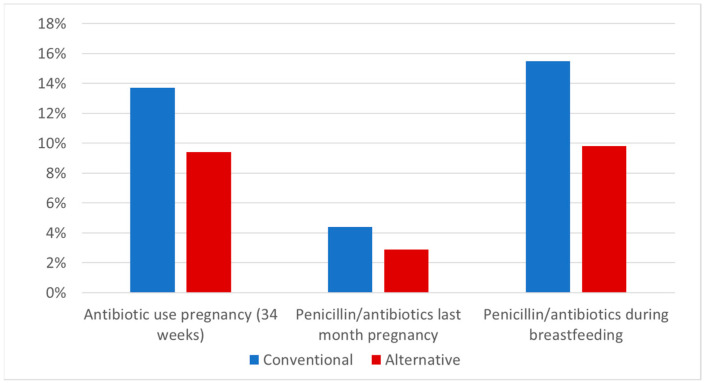
Antibiotic use by mothers during pregnancy and breastfeeding (when infants were 3 months old). Data on antibiotic use during pregnancy and in the last month of pregnancy were available for 2650 women (476 in the alternative lifestyle group and 2174 in the conventional lifestyle recruitment group) and during breastfeeding data were available for 2654 women (477 and 2177).

As shown in Figure 1, several significant differences between the two groups were found for antibiotics use by pregnant women and by children during the first 12 months of life. During pregnancy, 9.4% (46/489) of the participants in the alternative lifestyle group and 13.7% (321/2337) of the participants in the conventional lifestyle group used antibiotics (*p* = 0.026). In the last month of pregnancy, the antibiotics use was lower (2.9% vs. 4.4%) and the difference between the two groups was not statistically significant (*p* = 0.144). After delivery, a significant difference was found between both groups in the use of penicillin or other antibiotics during breastfeeding (*p* = 0.003): within the alternative group, 9.8% (44/447) of the breastfeeding women used antibiotics; in the conventional group, 15.5% (252/1631) did so.

Given the distinct maternal demographic and lifestyle characteristics in the alternative and conventional lifestyle groups (see Table 1), we set out to evaluate whether some of these characteristics could at least in part explain the differences in antibiotics consumption during pregnancy and lactation (see Table 3). During pregnancy, the difference between use of antibiotics in the two groups remained statistically significant even when maternal education and organic food choice were included. This was found to be true even though maternal education per se was associated with more frequent antibiotic use (OR, 1.53; 95% CI, 1.13–2.09). The difference between antibiotic use during lactation in the two groups remained statistically significant even when maternal education was included in the logistic regression model. Finally, it remained significant when including delivery mode (artificial vs. vaginal at home) and the number of older siblings. Maternal education per se was not associated with different antibiotic use during lactation.

**Figure 2:**
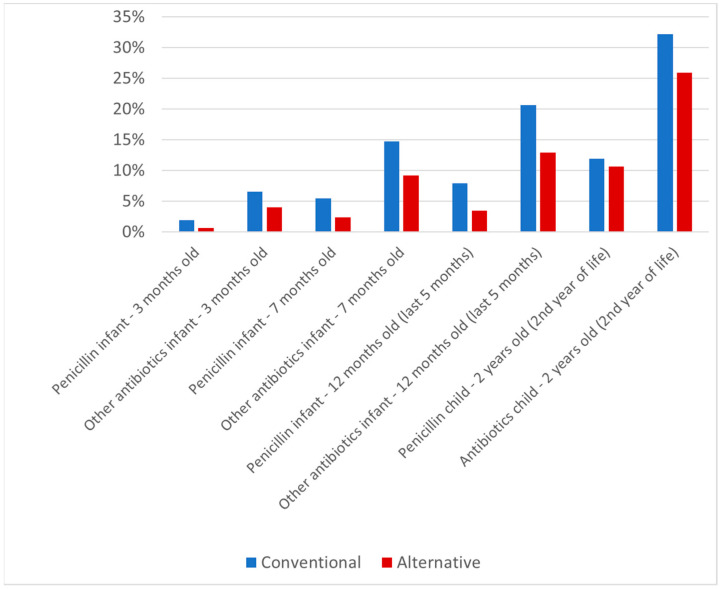
Antibiotic use in infants/children from 3 months to 2 years of age. Data on penicillin at 3 months of age were available for 2634 children (473 in the alternative lifestyle group and 2161 in the conventional lifestyle recruitment group); data on other antibiotics at 3 months of age were available for 2650 children (476 and 2174, respectively); data on penicillin at 7 months of age were available for 2588 children (471 and 2117); data on other antibiotics at 7 months of age were available for 2587 children (471 and 2116); data on penicillin at 12 months of age were available for 2549 children (467 and 2082); data on other antibiotics at 12 months of age were available for 2534 children (464 and 2070); data on penicillin at 2 years of age were available for 2461 children (435 and 2026); and data on other antibiotics at 2 years of age were available for 2512 children (448 and 2064).

From the age of 3 months to 2 years, antibiotic use increased in both groups (see Figure 2). Furthermore, the differences in antibiotic use between the alternative and the conventional groups also increased with age. Within the alternative group, statistically significantly fewer antibiotics were used than in the conventional group at all time-points except in the case of penicillin at 3 months of age (*p* = 0.052). The difference in antibiotic use between the conventional and alternative groups was relatively small between 3 and 7 months of age (1.3%; 2.5%; 3.1%), but it increased over time from 7 months to 2 years of age (5.6%; 4.5%; 7.7%; 6.9%; see Figure 2).

We reasoned that demographic and lifestyle characteristics of the mother could also be associated with antibiotic treatments in the case of small children. Antibiotic use by children was lower in the alternative than the conventional group in all periods covered by the questionnaires (see Table 4). In the univariable regression, this manifested as lower ORs with 95% CIs that excluded 1.0 (see Table 4), with the strongest association in the first year (ORs about 0.5). When adjusted in multivariable models, the association attenuated somewhat (to 0.6), but the 95% CIs still excluded 1.0, indicating that the other factors only partially explained the difference in antibiotic use between the alternative and conventional lifestyle groups. Maternal education and age were unrelated to antibiotic use. Risk factors for antibiotic use in the first year were hospital delivery (vs. home delivery, which had the strongest association for antibiotic use in the 8–12 month period) and having one or more older siblings; while breastfeeding duration showed a protective association (see Table 4). For antibiotic use in the second year, all these factors had no impact any more, while the difference between the alternative and conventional groups was retained, albeit in a less pronounced manner than in the first year (OR: 0.72). Anthroposophic orientation and use of organic foods were unrelated to children’s antibiotic use in multivariate models and did not change the difference between the alternative and conventional groups, and they were omitted from the final models.

In this section we report the results from the logistic regression with the child’s antibiotic use (AB) as the outcome variable (yes/no) in the periods covered by the questionnaires. The numbers of subjects may differ slightly from the baseline table due to a few missing values in covariables. ORs with 95% CI were used. Statistically significant results are printed bold. Breastfeeding was included as a continuous variable in months up to the end of the age period, e.g., for age periods 4–7 breastfeeding ranged from 0 (never breastfed) to 7 months (breastfeeding up to and including the 7th month). The ORs were multiplicative; for instance, the OR of 0.92 per month of breastfeeding translates to 0.92^3 = 0.78 for a contrast between 3 and 0 months. The same is true for a contrast between 7 and 3 months of breastfeeding, which would agree with about a 20% lower risk of antibiotic use, and 0.92^7 = 0.56 for a contrast between 7 and 0 months of breastfeeding, agreeing with about a 40% lower risk of antibiotic use (the logistic regression model assumes the linearity of the natural log of the odds).

**Figure 3:**
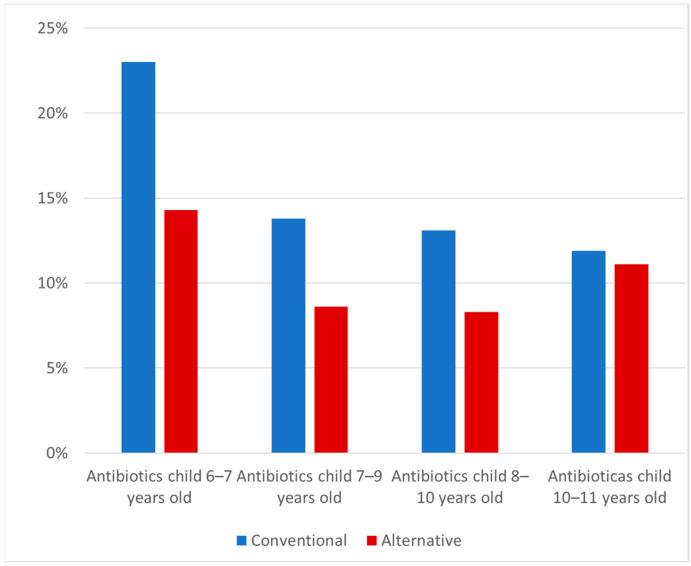
Antibiotic treatment of 6 to 11 year old children (in each case, most recent year). Data on antibiotics use at 6–7 years of age were available for 1964 children (362 in the alternative lifestyle group and 1602 in the conventional lifestyle recruitment group); at 7–9 years data were available for 1881 children (350 and 1531); at 8–10 years of age data were available for 1821 children (340 and 1481); and at 10–11 years of age data were available for 1634 children (314 and 1320).

From the age of 6 to 11 years, the number of antibiotic treatments decreased with age in both lifestyle groups, even though, within the alternative group, a slight increase in antibiotic use could be observed for the ages of 8–11 years (see Figure 3). Fewer children in the alternative lifestyle group than in the conventional group underwent antibiotic treatments. In most cases, the difference in antibiotic treatment between the two groups proved to be significant. This difference was relatively large at the age of 6–7 years and decreased as the children got older (8.6%; 5.2%; 4.9%; 0.8%).

**Figure 4:**
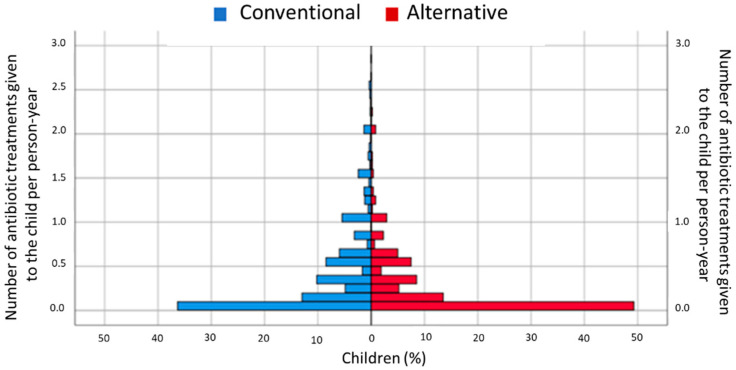
Number of antibiotic treatments given to a child per person-year. The number of antibiotic treatments per person-year was calculated by dividing the sum of all antibiotic treatments by observation time. From 3 months to 12 months, information was available on use or non-use of antibiotics; if the parents reported any antibiotic treatment, 1 antibiotic course was assumed. Thereafter, the number of antibiotic courses (1, 2, 3 or more antibiotic courses) was asked for directly. The time consisted of the number of months addressed in the successive questionnaires (from data from previous questionnaire to that of the current questionnaire). In this way, it was possible to control for differences in the timing of filling in the various questionnaires.

Figure 4 shows that, in the two lifestyle groups, the majority of the children had no antibiotics treatments (i.e., 0 = zero antibiotic treatments per person-year), but it also reveals several differences between the two lifestyle groups. Firstly, the percentage of children with 0 antibiotic treatments per person-year was higher in the alternative group than in the conventional group. In addition, the percentages of children with between 1–2 antibiotic treatments and 2–3 antibiotic treatments per year were lower in the alternative lifestyle group than they were in the conventional lifestyle group.

## 3. Discussion

Taken together, our data show that pregnant and breastfeeding women, as well as children, in the alternative lifestyle group generally used antibiotics less often than in the conventional lifestyle group. Interestingly, the differences between the two groups with regard to maternal antibiotic use were significant during pregnancy and breastfeeding and continued in their offspring from the beginning, increasing up to age 6–7. Thereafter, the difference was attenuated. Use of penicillin followed the pattern of the remaining antibiotics, except at age two, when use of penicillin was similar in both lifestyle groups. Among the children, the number of antibiotic treatments per person-year was lower in the alternative group than in the conventional group.

The KOALA Birth Cohort Study is being carried out in the Netherlands, a country with one of the lowest frequencies of antibiotics consumption worldwide [12]. According to OECD data, average antibiotic consumption in the Netherlands was about 12 defined daily doses per day (DDD) per 1000 inhabitants in 2014, which was approximately half of the average OECD consumption; no increase was observed between 2005 and 2014 [12]. The fact that lifestyle influences antibiotics consumption suggests that even in the Netherlands a considerable portion of the prescriptions are not really needed. This is in line with results showing that in the Netherlands approximately 50% of antibiotic prescriptions for bronchitis, tonsillitis and sinusitis were not issued in accordance with guidelines [13]. It appears conceivable that, in countries with higher consumption, stronger effects from lifestyle can be expected. Interestingly, the lower consumption of antibiotics was already apparent during pregnancy (30% fewer antibiotic treatments in the alternative lifestyle group), a period in which decisions have to be particularly carefully considered, as infections may negatively affect the course of pregnancy, as well as mothers’ and children’s health [14], at the same time that any unnecessary treatment should be avoided. The lower consumption of antibiotics during pregnancy in the alternative lifestyle group relative to the conventional group could in part be associated with the lower prevalence of urinary infections in that group. The similar (or even higher) frequency of the remaining infectious diseases and fever in the alternative group speaks against such associations however. Finally, the obstetric outcomes, duration of pregnancy and birth weight were comparable between the two groups, suggesting that the (30%) lower antibiotic use did not have negative consequences. 

The main limitation of the present analysis was the lack of information on children’s health outcomes and complications/hospitalizations. Moreover, it would also have been interesting to add information on how physicians who were consulted by participants in the two recruitment groups worked; for instance, how much time they invested in patient care, how many patients they saw per day and how much communication with the parents/caregivers occurred per infectious syndrome.

The higher educational level of the alternative group mothers could have contributed to the lower antibiotic use for themselves and among their children. A Danish cohort study showed that the prevalence of antibiotic use was higher among mothers with a lower level of education [15]. Similarly, a survey on parental knowledge, attitudes and practice of antibiotic treatment in children found that low parental education was the most important independent risk factor positively related to antibiotic use [16]. Our results confirm that lower education is associated with higher antibiotic use during pregnancy (but not during lactation). Differences in maternal education (which can also be taken as an income indicator) did not, however, explain the observed differences between antibiotic treatment during pregnancy—nor during lactation—in the alternative and conventional lifestyle groups. Differences in antibiotic use during lactation between the two lifestyle groups were due neither to distinct maternal education levels nor to delivery mode (artificial vs. vaginal at home) or number of older siblings (as an indicator for maternal experience). 

With regard to early childhood and in line with previous studies [17], our results corroborated that breastfeeding protects against infections in the first year of life. Moreover, they revealed more frequent antibiotic treatments in children delivered in hospital (relative to home deliveries that are usual in the Netherlands), which may be related to medicalization (e.g., parents being more inclined to seek medical care), but perhaps also to a higher risk of breastfeeding problems (especially after cesarean operations). Older siblings were included as an indicator of maternal experience with child care (expected to lead to, e.g., more non-antibiotic home medication for a feverish child) but the results indicated a higher antibiotic use (likely due to older siblings being a source of infections). Finally, the alternative lifestyle group included a considerable proportion of families with an anthroposophic orientation and users of organic foods (Table 1). Adherence to anthroposophy is likely to be associated with–but not *conditio sine qua non* for–the use of anthroposophic medicine. This type of medicine (see below) advocates prudent use of childhood vaccination and antibiotic use to favor the development of natural immunity to infections in children [18]. However, the association between adherence to anthroposophy and antibiotic use was weak in our study. Also, organic food consumption during pregnancy (as indicator of investment in prudent or nature-oriented lifestyles) was not associated with antibiotic use. Neither adherence to anthroposophy not organic food consumption explained the lower use in the alternative group. Other lifestyle characteristics that strongly influence antibiotic use—be it during pregnancy, lactation or early childhood—deserve further investigations.

Given that the decision for or against an antibiotic treatment should be taken exclusively by physicians, influences of parental lifestyle are at first sight surprising. However, patient expectations appear to be a strong predictor of general practitioners’ prescribing behavior [19] and patients’ expectations and physicians’ assumptions regarding these expectations are—next to diagnostic uncertainty—important factors leading to over-prescription of antibiotics [20,21,22]. This suggests that physicians have to be trained not only to prescribe fewer antibiotics, but also to communicate the disadvantages of unnecessary use of antibiotics to lay people, namely to their patients and, in the case of child patients, their parents. Especially in situations in which parents are anxious, as frequently happens when children have a fever and specific symptoms, is it important that physicians are able to deliver consistent and reliable information [21]. In addition, simple measures, such as introducing an illness-focused interactive booklet, might have positive effects [23]. Since the knowledge and attitude of patients concerning antibiotics can possibly influence physicians’ expectations [19], their improvement should also be a goal of public health measures. It should be kept in mind that interventions aimed at reducing the intentions to use antibiotics among of in child-bearing age may contribute to slowing down the development of antibiotic resistance and reducing the negative side effects of antibiotics in children.

In principle, the higher adherence to life rules—related to religious or philosophical backgrounds—in the alternative lifestyle group might have contributed to the lower antibiotics use in this group. This might have happened directly if their rules included restrictions on antibiotics, but also indirectly if the rules strengthened the community and the parents who belonged to this community had specific expectations from therapies other than antibiotics. In the alternative lifestyle group, the number of participants who reported adhering to an anthroposophic lifestyle was higher than in the conventional lifestyle group and the large majority of the participants mentioned choosing products from—anthroposophy-inspired—biodynamic production. Therefore, and also due to the means of recruitment (in part via anthroposophic general practitioners and midwifes, anthroposophic under-five clinics and Rudolf Steiner schools), it is likely that more women and children in the alternative lifestyle group were being treated by physicians with a training in anthroposophic medicine. This type of integrative medicine [24,25] promotes a restricted use of antibiotics at the same time that supportive treatments are developed and actively used. Especially in respiratory and ear infections—two very frequent indications for antibiotic use in children—anthroposophic physicians tend to prescribe fewer antibiotics [26]. In both types of infections, there is a broad discussion on the advantages of watchful waiting (see [27] and [28], respectively). At least in the case of some types of otitis media, a multimodal approach that includes several non-invasive supportive therapies (pneumatization exercises, education, an antiallergenic diet, nasal hygiene, useful constitutional therapy and thermal interventions; e.g., P.E.A.N.U.T.) appears to contribute to the success of waiting [29]. The fact that physicians with training in anthroposophic medicine hold longer consultations than is usual in conventional medicine (see [30]) could also contribute to taking the risk of not prescribing antibiotics right away. Finally, it is conceivable that further therapies from complementary medicine may contribute to reducing unnecessary antibiotics consumption [31].

To achieve a global reduction of antibiotics use, it is necessary to know how to tailor the required scientific information and the corresponding recommendations to various countries and even regions [32]. By emphasizing the importance of co-existing parental lifestyles in the use of antibiotics during pregnancy and during the first years of their offspring’s lives, our work adds to the ongoing discussion. Information and recommendations should also take aspects of lifestyle into consideration. A more detailed—possibly qualitative—investigation of the reasons behind the restrictive use of antibiotics associated with alternative lifestyles might unveil new possibilities for improving motivation in the general population.

## 4. Materials and Methods

### 4.1. Study Population

The KOALA Birth Cohort Study is an ongoing cohort study in the Netherlands of factors influencing atopic disease, with a main focus on lifestyle [18]. The study was approved by the Medical Ethics Committee of Maastricht University Medical Centre, the Netherlands, and written informed consent was given by the parents of the participating children. From October 2000 to December 2002, a total of 3030 pregnant women were recruited at 34 weeks of pregnancy. Pregnant women with a conventional lifestyle (n = 2343) were recruited from an ongoing prospective cohort study on pregnancy-related pelvic pain in the Netherlands [18]. The other group of pregnant women (n = 491) were recruited through alternative lifestyle-related channels, such as organic food shops, anthroposophic general practitioners and midwives, anthroposophic under-five clinics, Rudolf Steiner schools and relevant magazines [18].

### 4.2. Data on Antibiotic Use

Information on antibiotic use was collected by repeated parent questionnaires from 34 weeks of pregnancy to 11 years of age, in which different types of questions on antibiotic use were asked. Whether or not the mother used antibiotics during pregnancy or while breastfeeding and whether the infant was given antibiotics until 3 months of age was analyzed as a categorical variable (“no”, “yes”, “don’t know”). From 3 months of age to 1 year of age, whether or not the child was given penicillin or other antibiotics and for how long were analyzed using categorical variables (“no”, “yes 1–3 days”, “yes 4–10 days”, “yes more than 10 days”). These variables were chosen to split the group into “no” and “yes” groups, where the category “yes” contained the three response options (1–3 days, 4–10 days and more than 10 days). At the age of 2 years, the use of penicillin or other antibiotic treatment during the child’s second year of life, as well as how often this occurred, was surveyed by means of categorical variables (“never”, “once”, “twice”, “more than twice”, “don’t know”). The number of antibiotic courses prescribed to children from the age of 6 to 11 years was also analyzed as a categorical variable (“never”, “once”, “twice”, “three times or more”). From 3 months of age on, the initial text of the questions on antibiotics treatment was “How often did your child (have) one of the following medications in … [observational time]?”; thereafter, a distinction was made between treatment with penicillin and treatment with other antibiotics. The text on penicillin-only treatments was: “Penicillin for strep throat or skin infection (syrup/drink, such as: Broxil [the acid-stable penicillin derivative pheneticillin], Acipen-V [phenoxymethylpenicillin], Phenoxymethyl-penicillin)”; the text on other antibiotics was: “Antibiotic treatment for other infections or inflammations such as ear infection, bladder infection etc. (drops/suspension, such as Clamoxyl [amoxicillin], Amoxi Disp [amoxicillin dispersible tablets], Amoxicillin, Flemoxin [amoxicillin], Zithromax [azithromycin], Doxycyline, Co-trimoxazole [trimethoprim/sulfamethoxazole], Bactrimel [trimethoprim], Eusaprim [trimethoprim/sulfamethoxazole], Trimethoprim, Monotrim [trimethoprim], Sulphamethizole, Sulphafurazole, Sulfadiazine)”.

### 4.3. Statistical Analysis

Descriptive data on the alternative and conventional groups were analyzed using descriptive statistics with the IBM SPSS Statistics 25 statistical software; the results were presented as numbers and percentages. The baseline measurement was available for 2834 respondents in total, of whom 2343 were from the conventional lifestyle recruitment group and 491 from the alternative lifestyle recruitment group. Numbers and percentages of the two groups were compared by categorical variables on the use of antibiotics during pregnancy, breastfeeding and oral treatment of the child. In total, 18 outcome variables related to antibiotic use were analyzed. Furthermore, several demographic, lifestyle and health-related characteristics of the alternative and conventional recruitment groups were analyzed as independent determinants.

Differences between both recruitment groups were evaluated by Chi-square test and analysis of variance for continuous and categorical variables, respectively. Statistical significance was set at *p* < 0.05. Logistic regression was used to estimate the OR for antibiotic use, comparing the alternative and conventional lifestyle groups, without and with adjustment for the possible confounders. The KOALA cohort study has a long follow-up period and not all respondents participated in the study for the same amount of time. To take this into account, person-years were used to calculate the number of antibiotic treatments given to the respondents. For the period from 3 months to 12 months, one antibiotic treatment was assumed; for children aged 2 years to 11 years, the number of antibiotic treatments (1, 2 or 3 or more antibiotic treatments) was available. The time period corresponding to the antibiotic intake consisted of the number of months until the questionnaire on the child’s antibiotic use had been returned.

## Figures and Tables

**Table 1:** Demographic and lifestyle characteristics of the mothers from the two recruitment groups.

Characteristics		Alternative Lifestyle Group (N = 491)		Conventional Lifestyle Group (N = 2343)	
		n/N or n	% or Mean ± S.D.	n/N or N	% or Mean ± S.D.
**Age of mother**		491	33.7 ± 4.3	2340	31.7 ± 3.6
**Highest educational attainment**	WO Academic education	132/491	27.0%	256/2340	11.1%
	HBO Higher vocational education	225/491	46.0%	728/2340	31.5%
	MBO Vocational secondary education	52/491	10.6%	706/2340	30.6%
	VWO—high school Pre-university education	18/491	3.7%	57/2340	2.5%
	HAVO—high school Senior general secondary education	33/491	6.8%	194/2340	8.4%
	MAVO—high school Pre-vocational secondary education	8/491	1.6%	145/2340	6.3%
	LBO—high school Lower vocational secondary education	8/491	1.6%	123/2340	5.3%
	Elementary school	2/491	0.4%	3/2340	0.1%
	Other	11/491	2.3%	97/2340	4.2%
	Missing values	2/491	0.4%	34/2340	1.5%
**Adhered to living rules during the last month**		156/491	32.6%	51/2340	2.2%
**Adhered to…**					
**...Vegetarianism**		83/491	16.9%	36/2343	1.5%
**…Veganism**		1/491	0.2%	1/2343	0.0%
**…Macrobiotics**		6/491	1.2%	1/2343	0.0%
**…Anthroposophic lifestyle**		70/491	14.3%	3/2343	0.1%
**…Life reform movement**		19/491	3.9%	4/2343	0.2%
**…Islam**		0/491	0.0%	10/2343	0.4%
**…Buddhism**		3/491	0.6%	0/2343	0.0%
**…Judaism**		3/491	0.6%	0/2343	0.0%
**…Hinduism**		0/491	0.0%	1/2343	0.0%
**…Other**		31/491	6.3%	6/2343	0.3%
**Intentional choice of some organic products**					
**Consumed organic products**		428/490	87.3%	485/2333	20.8%
**Did not consume organic products**		52/490	10.6%	1471/2333	63.1%
**Did not know/did not care**		10/428	2.0%	377/2333	16.2%
**Intentional choice of biodynamic products**					
**Consumed biodynamic products**		357/488	73.2%	167/2327	7.2%
**Did not consume biodynamic products**		131/488	26.8%	2120/2327	92.8%

**Table 2:** Health-related characteristics of the mothers from the two recruitment groups.

Characteristics	Alternative Lifestyle Group (N = 491)		Conventional Lifestyle Group (N = 2343)		
	n/N or n	% or Mean ± S.D.	n/N or N	% or Mean ± S.D.	*p*-Value
**Candida infection in the mouth during the pregnancy**	6/490	1.2%	13/2339	0.6%	0.095
**Candida infection in the genital area during the pregnancy**	111/488	22.7%	330/2340	14.1%	<0.001
**Aphthae during the pregnancy**	63/489	12.9%	186/2338	8.0%	0.001
**Lip cold sores during the pregnancy**	58/487	11.9%	287/2338	12.3%	0.446
**Common cold after third month**	315/490	64.3%	1502/2339	64.2%	0.510
**Fever after third month**	46/490	9.4%	222/2338	9.5%	0.510
**Flu after third month**	52/491	10.6%	252/2337	10.8%	0.488
**Diarrhea after third month**	97/490	19.8%	551/2334	23.6%	0.037
**Urinary tract infections after third month**	28/489	5.7%	187/2338	8.0%	0.048
**Duration of pregnancy (weeks)**	480	39.6 ± 1.4	2332	39.5 ± 1.5	0.128
**Birth weight (g)**	482	3582 ± 506	2330	3487 ± 512	<0.001

**Table 3:** Antibiotic use (AB) during pregnancy and lactation as a function of recruitment groups and selected demographic and lifestyle characteristics.

	During Pregnancy AB (%) Total	During Lactation AB (%) Total
**Antibiotic use**		
Conventional group	321 (13.8%) 2327	252 (15.5%) 1629
Alternative group	46 (9.4%) 488	44 (9.8%) 447
**Univariable model**	OR (95%CI)	OR (95%CI)
Conventional group	1.0 (reference)	1.0 (reference)
Alternative group	**0.65 (0.47–0.90)**	**0.60 (0.42–0.84)**
**Multivariable models**		
Conventional group	1.0 (reference)	1.0 (reference)
Alternative group	**0.67 (0.48–0.94)**	**0.63 (0.44–0.90)**
High education	1.0 (reference)	1.0 (reference)
Mid-level education	1.13 (0.88–1.45)	0.81 (0.61–1.08)
Low education	**1.59 (1.17–2.18)**	0.98 (0.66–1.45)
Home delivery	-	1.0 (reference)
Natural hospital birth	-	1.12 (0.84–1.50)
Instrumental hospital *	-	**1.43 (1.02–2.00)**
No older siblings	-	1.0 (reference)
One or more siblings	-	0.91 (0.61–1.21)
Breastfeeding <1 month	-	1.0 (reference)
Breastfeeding 1 month	-	1.22 (0.70–2.11)
Breastfeeding 2 months	-	1.00 (0.60–1.66)
Breastfeeding 3 months	-	0.95 (0.60–1.49)
Maternal age (per year)	1.03 (0.99–1.06)	0.99 (0.96–1.03)

* instrumentally assisted delivery and cesarean section. Results from logistic regression with the maternal antibiotic use (AB) as the outcome variable (yes/no). The analysis of AB use during lactation was confined to mothers who had initiated breastfeeding; numbers of subjects may further differ slightly from the baseline table due to a few missing values in covariables. Odds ratios (ORs) with 95% confidence intervals (95% CIs) were used in the logistic regression analysis, with antibiotic use as the outcome variable and a univariable model with only the alternative vs. conventional groups and with the indicated multivariable adjustment. Statistically significant results (*p* < 0.05) are printed bold.

**Table 4:** Antibiotic treatment (AB) of children as a function of recruitment groups and selected demographic and lifestyle characteristics.

		Age Period (Children)		
	0–3 months AB (%) total	4–7 months AB (%) total	8–12 months AB (%) total	2nd year AB (%) total
**Antibiotic use**				
Conventional group	169 (7.8%) 2161	397 (18.8%) 2117	548 (26.3%) 2085	519 (25.9%) 2003
Alternative group	20 (4.2%) 473	49 (10.4%) 472	71 (15.2%) 467	86 (19.6%) 439
**Univariable model**	OR (95%CI)	OR (95%CI)	OR (95%CI)	OR (95%CI)
Conventional group	1.0 (reference)	1.0 (reference)	1.0 (reference)	1.0 (reference)
Alternative group	**0.52 (0.32–0.84)**	**0.50 (0.37–0.69)**	**0.50 (0.38–0.66)**	**0.70 (0.54–0.90)**
**Multivariable models**				
Conventional group	1.0 (reference)	1.0 (reference)	1.0 (reference)	1.0 (reference)
Alternative group	**0.60 (0.36–0.99)**	**0.63 (0.45–0.88)**	**0.59 (0.44–0.79)**	**0.72 (0.54–0.95)**
High education	1.0 (reference)	1.0 (reference)	1.0 (reference)	1.0 (reference)
Mid-level education	0.99 (0.70–1.40)	0.84 (0.66–1.06)	0.88 (0.71–1.08)	1.05 (0.85–1.29)
Low education	1.31 (0.86–2.02)	0.90 (0.66–1.24)	0.92 (0.69–1.23)	1.04 (0.78–1.39)
Home delivery	1.0 (reference)	1.0 (reference)	1.0 (reference)	1.0 (reference)
Natural hospital birth	1.40 (0.99–1.97)	1.13 (0.89–1.43)	**1.34 (1.08–1.66)**	1.16 (0.94–1.43)
Instrumental hospital	1.44 (0.94–2.19)	1.14 (0.85–1.53)	**1.62 (1.25–2.09)**	1.08 (0.83–1.41)
No older siblings	1.0 (reference)	1.0 (reference)	1.0 (reference)	1.0 (reference)
One or more siblings	**1.57 (1.11–2.23)**	**1.45 (1.14–1.83)**	**1.33 (1.07–1.64)**	0.90 (0.73–1.10)
Breastfeeding (per month)	0.92 (0.84–1.02)	**0.92 (0.88–0.95)**	**0.95 (0.93–0.98)**	0.99 (0.97–1.02)
Maternal age (per year)	0.98 (0.94–1.02)	0.98 (0.95–1.01)	1.01 (0.98–1.04)	1.01 (0.99–1.04)

## Data Availability

All data generated or analyzed during the current study are available from the corresponding author on reasonable request.
